# Supplementary material for: Monitoring of Unfractionated Heparin Therapy in the Intensive Care Unit Using a Point-of-Care aPTT: A Comparative, Longitudinal Observational Study with Laboratory-Based aPTT and Anti-Xa Activity Measurement
Source: J Clin Med. 2022 Feb 28;11(5):1338. doi: 10.3390/jcm11051338 (PMC8911237; doi:10.3390/jcm11051338)
Supplement: Supplementary file 1 [file jcm-11-01338-s001.zip › Table S1.pdf]

Table S1. Results of individual regression models.

Variable	Coefficient	p-value	Interaction coefficient	p-value
CRP, mg/dL	−0.025 (−0.06; 0.007)	0.17	0.001 (0.0004; 0.002)	**0.006**
Fibrinogen, g/L	0.007 (−0.006; 0.02)	0.23	−0.0002 (−0.0005; −0.000003)	**0.04**
Factor VIII, %	0.03 (−0.003; 0.06)	0.11	−0.0008 (−0.001; −0.0003)	**0.006**
Factor XI, %	0.19 (0.07; 0.33)	**0.003**	−0.009 (−0.01; −0.006)	**<0.001**
Factor XII, %	0.07 (−0.11; 0.25)	0.44	−0.006 (−0.01; −0.003)	**<0.001**
Antithrombin, %	−0.10 (−0.15; −0.05)	**<0.001**	**-**	ns
Lupus anticoagulant, positive	−6.22 (−12.86; −0.48)	**0.04**	0.19 (0.03; 0.34)	**0.02**

Results of individual linear mixed-effects models including POCT-APTT as the dependent variable and lab-APTT plus each potential confounding variable individually as fixed independent variables. Coefficients for the variable and its interaction term with lab-APTT are given for each parameter with the associated p-value. A significant coefficient means that the variable induced a systematic difference between POCT- and lab-APTT. A significant interaction term means that the variable modified the correlation between POCT- and lab-APTT.

CRP, C-reactive protein; ns, not significant.
