# Supplementary material for: Monitoring of Unfractionated Heparin Therapy in the Intensive Care Unit Using a Point-of-Care aPTT: A Comparative, Longitudinal Observational Study with Laboratory-Based aPTT and Anti-Xa Activity Measurement
Source: J Clin Med. 2022 Feb 28;11(5):1338. doi: 10.3390/jcm11051338 (PMC8911237; doi:10.3390/jcm11051338)
Supplement: Supplementary file 1 [file jcm-11-01338-s001.zip › Table S2.pdf]

Table S2. Results of the final multivariable model.

Variable	Coefficient	p-value
Intercept, s	10.53<br>(−4.59; 25.48)	
Lab-APTT, s	1.07<br>(0.77; 1.36)	**<0.001**
CRP, mg/dL	−0.05<br>(−0.09; −0.006)	**0.04**
Interaction between<br>CRP & Lab-APTT, s.mg/dL	0.002<br>(0.0007; 0.002)	**0.001**
Fibrinogen, g/L	0.02<br>(−0.002; 0.04)	0.07
Interaction between<br>Fibrinogen & Lab-APTT, s.g/dL	−0.0006<br>(−0.0009; −0.0002)	**<0.001**
FXII, %	−0.01<br>(−0.21; 0.18)	0.89
Interaction between<br>FXII & Lab-APTT, s.%	−0.004<br>(−0.008; −0.0004)	**0.03**
Lupus anticoagulant, positive	−6.15<br>(−13,26; 2.45)	0.11
Interaction between<br>Lupus anticoagulant & Lab-APTT, s	0.20<br>(0.005; 0.39)	**0.04**

Results of the final multivariable linear mixed-effects model including POCT-APTT as the dependent variable and lab-APTT plus significant confounders as fixed independent variables. Coefficients of the variables and interactions termd are given with the associated p-values. A significant coefficient means that the variable induced a systematic difference between POCT- and lab-APTT. A significant interaction term means that the variable changed the correlation between POCT- and lab-APTT.

APTT, activated partial thromboplastin time; CRP, C-reactive protein.
