# Supplementary material for: Monitoring of Unfractionated Heparin Therapy in the Intensive Care Unit Using a Point-of-Care aPTT: A Comparative, Longitudinal Observational Study with Laboratory-Based aPTT and Anti-Xa Activity Measurement
Source: J Clin Med. 2022 Feb 28;11(5):1338. doi: 10.3390/jcm11051338 (PMC8911237; doi:10.3390/jcm11051338)
Supplement: Supplementary file 1 [file jcm-11-01338-s001.zip › Table S3.pdf]

Table S3. Comparison of the different tests used to monitor unfractionated heparin therapy.

	**Point-of-care testing APTT** (CoaguChek® Pro II, Roche)	**Laboratory-based APTT** (C.K. Prest®, Stago)	**Laboratory-based Anti-Xa Activity** (STA®Liquid anti-Xa, Stago)
Time to results (minutes)	< 5	50.9 (38.4-69.1)	66.3 (49.0-91.8)
Global TAT from blood collection until dose adjustment (minutes)	Not evaluated	92.0 (69.3-121.2)	Not evaluated
Sample matrix (minimal volume required)	A drop of fresh whole blood (> 8µl)	3.2% buffered citrated plasma (>250 µl)	3.2% buffered citrated plasma (>250µl)
Analysis principle	Electrochemical measurement of the APTT using celite as activator.	Coagulometric measurement of the APTT using kaolin as activator	Chromogenic assay without antithrombin supplementation
Measurement of UFH Activity	Indirect	Indirect	Direct
Intra and inter-assay CV (%)	2.1 – 5.1*	0.7 – 1.7	3.3 – 4.4
LLoQ	20 seconds	20 seconds	0.1 IU/ml
Disadvantages	Poor correlation to Anti-Xa activity and affected by CRP in multivariable model;<br>Costs	Affected by many preanalytical and analytical variables;<br>Age-dependent effect of UFH and baseline APTT	Underestimation in case of severe antithrombin deficiency;<br>Cost;<br>Experienced staff
Benefits	Time savings;<br>Low analyzed volume;<br>Fresh drop of whole blood	Availability and familiarity of clinicians;<br>Low cost;<br>Detectable factor deficiencies or DIC if issuing heparinase	Specific to heparin activity

Times are expressed as median (Interquartile range)
*Obtained from the manufacturer's kit insert.

APTT, activated partial thromboplastin time; CRP, C-reactive protein; LLoQ, lower limit of quantification; TAT, turnaround time; UFH, Unfractionated heparin
